# Macrophage Migration Inhibitory Factor Alters Functional Properties of CA1 Hippocampal Neurons in Mouse Brain Slices

**DOI:** 10.3390/ijms21010276

**Published:** 2019-12-31

**Authors:** Eric Bancroft, Rahul Srinivasan, Lee A. Shapiro

**Affiliations:** Department of Neuroscience and Experimental Therapeutics, Texas A&M University Health Science Center, College of Medicine, Bryan, TX 77807, USA; eric369@exchange.tamu.edu

**Keywords:** neuroinflammation, neurotrauma, traumatic brain injury (TBI), ischemic stroke, epilepsy, macrophage migration inhibitory factor (MIF)

## Abstract

Neuroinflammation is implicated in a host of neurological insults, such as traumatic brain injury (TBI), ischemic stroke, Alzheimer’s disease, Parkinson’s disease, and epilepsy. The immune response to central nervous system (CNS) injury involves sequelae including the release of numerous cytokines and chemokines. Macrophage migration inhibitory factor (MIF), is one such cytokine that is elevated following CNS injury, and is associated with the prognosis of TBI, and ischemic stroke. MIF has been identified in astrocytes and neurons, and some of the trophic actions of MIF have been related to its direct and indirect actions on astrocytes. However, the potential modulation of CNS neuronal function by MIF has not yet been explored. This study tests the hypothesis that MIF can directly influence hippocampal neuronal function. MIF was microinjected into the hippocampus and the genetically encoded calcium indicator, GCaMP6f, was used to measure Ca^2+^ events in acute adult mouse brain hippocampal slices. Results demonstrated that a single injection of 200 ng MIF into the hippocampus significantly increased baseline calcium signals in CA1 pyramidal neuron somata, and altered calcium responses to *N*-methyl-d-aspartate (NMDA) + D-serine in pyramidal cell apical dendrites located in the stratum radiatum. These data are the first to show direct effects of MIF on hippocampal neurons and on NMDA receptor function. Considering that MIF is elevated after brain insults such as TBI, the data suggest that, in addition to the previously described role of MIF in astrocyte reactivity, elevated MIF can have significant effects on neuronal function in the hippocampus.

## 1. Introduction

Traumatic brain injury (TBI) is a frequently occurring injury, with an annual incidence rate in the U.S. of greater than 1.7 million people. Approximately 5.3 million people in the U.S. live with TBI associated disability, resulting in an estimated annual economic burden of $48 billion [1]. Adolescent males, young children and the elderly, represent a large majority of TBI cases [1], although all demographics are affected. Treatments for TBI have thus far been lacking and all clinical trials have failed. Therefore, it is imperative to develop a better mechanistic understanding of TBI, so that therapeutic targets can be identified and TBI-associated impairments can be improved. 

A TBI results in two phases of injury; the primary injury being mechanical impact to the brain, while the secondary injury involves an early and often prolonged immune response. TBIs are highly variable and the primary injury can be mild sub-concussive, concussive or penetrating, each with a different degree of injury-related severity, and each impacting different areas of the brain, with different angular and rotational forces. Despite this variability, there are some relatively homogeneous features of TBI pathology, including the rapid induction of an immune response and a neuroinflammatory response to injury. 

The initial TBI-induced immune response is a non-specific innate response, which occurs rapidly after a TBI. In some cases, the initial innate immune response can precede an antigen-specific adaptive immune response [2]. The innate immune response to TBI involves a sequelae of events, including the release of damage-associated molecular patterns (DAMPs) and pathogen-associated molecular patterns (PAMPs) [3], which interact with pattern recognition receptors (PRRs). The PRRs include toll-like receptors (TLRs) and nucleotide-binding oligomerization domain-like (NOD) receptors (NLRs), that are expressed on neurons and glial cells, including astrocytes and microglial cells, as well as central nervous system (CNS)-infiltrating macrophages [4,5,6]. In many cases, the net effect of this receptor interaction is the production and subsequent release of cytokines and chemokines. Binding of DAMPs and/or PAMPs to PRRs, triggers a cellular signaling response involving multiple kinases, including NFκB kinase. NFκB levels are elevated following TBI and is associated with increases in numerous other cytokines (e.g., IL-6, TNF-a), as part of the progression of the innate inflammatory response [7]. 

Among the milieu of elevated inflammatory proteins after a CNS injury, is the cytokine, macrophage migration inhibitory factor (MIF) [8,9,10]. MIF, a 12.5 kD protein, is elevated after a TBI and the extent of this elevation is proportional with the prognosis of TBI, such that more severe injuries are associated with higher levels of MIF [8]. MIF is a secreted immunoregulatory cytokine that stimulates the inflammatory response, plays a role in responding to pathogens, and contributes to other immune or autoimmune events [11]. MIF is highly expressed in multiple brain regions, including the cortex, hypothalamus, hippocampus, cerebellum, and pons [12]. Most MIF within the CNS is thought to be synthesized locally, as high levels of MIF mRNA have been detected in astrocytes, including human astrocytes [13], and neuronal cell bodies [14]. Following CNS insults such as stroke, spinal cord injury and TBI, MIF concentration is elevated within cells, CSF and serum. MIF is also found to be elevated in numerous neuroinflammatory and neurodegenerative diseases, including: Multiple sclerosis, meningitis, encephalitis tick-borne borreliosis, subarachnoid hemorrhage, and Alzheimer’s disease [15,16,17,18,19,20,21,22,23,24,25,26,27]. Thus, MIF may be an important protein to examine as both a prognostic indicator of, and possible therapeutic target for TBI, numerous neuroinflammatory conditions, and Alzheimer’s disease.

Some of the functions of MIF in the immune system and brain have been previously explored. In an innate immune response, MIF binds to the CD74 receptor complex, along with co-stimulatory factor CD44, initiating downstream signaling cascades and facilitating the immune response [11,28,29,30,31,32,33,34,35,36,37,38,39,40]. The MIF/CD74 signaling complex is also conserved in microglia and astrocytes. In microglia, MIF/CD74 interaction inhibits microglial activation and leads to decreased levels of pro-inflammatory cytokines, such as IFN-γ [41]. Conversely, in astrocytes, MIF/CD74 interaction facilitates astrocyte reactivity, stimulates pro-inflammatory cytokine production, stimulates extracellular signal-related kinase (ERK) pathways, and increases NFκB levels [37,42,43]. In addition to these immunomodulatory roles, recent studies support the ability of MIF to modulate trophic functions in the CNS. MIF has been shown to directly bind and inhibit serine protease HTRA1 activity on FGF8 in astrocytes, which prevents the enzymatic breakdown of FGF8 and facilitates astrocyte migration [44]. Furthermore, 48 h of MIF exposure stimulated axonal growth [45], consistent with a trophic potential shared by numerous other cytokines and chemokines [46,47].

Numerous cytokines that have been shown to exhibit trophic functions, have also been found to directly modulate neuronal functioning, including synaptic plasticity and long-term potentiation of hippocampal neurons [46,47]. Consistent with this notion, MIF has been shown to inhibit Angiotensin II-elicited (AngII) increases in neuronal firing via interactions with the intracellular domain of AngII [48]. Moreover, evidence suggests that MIF might modulate the activity of neurons via glial cells, or directly modulate the firing frequency of neurons [45]. One recent study observed increased firing frequency in dorsal root ganglion (DRG) cells following acute bath application of the MIF peptide [45]. Since acute MIF application is sufficient to modulate neuronal excitability in the peripheral nervous system (PNS), similar mechanisms may exist in the CNS. Based on this rationale, we sought to determine if MIF can modulate hippocampal neuronal function. MIF was microinjected into the hippocampus and Ca^2+^ responses in CA1 pyramidal neurons from live mouse brain hippocampal slices expressing GCaMP6f were assessed. The results supported rejection of the null hypothesis and demonstrated that *in vivo* microinjection of MIF alters CA1 pyramidal neuron function.

## 2. Results

### 2.1. Macrophage Migration Inhibitory Factor (MIF) Alters Baseline Ca^2+^ Event Frequency in CA1 Pyramidal Neurons

**Figure 1:**
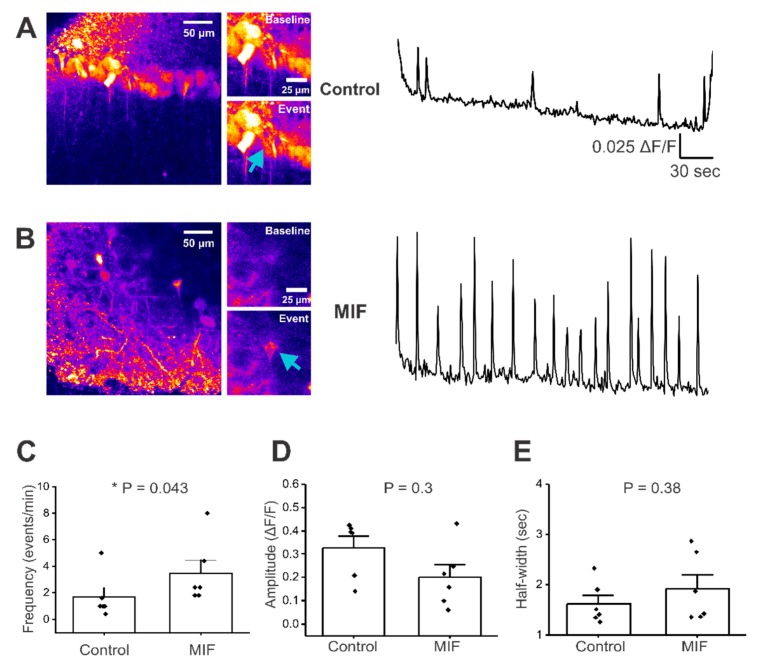
Baseline activity in CA1 neuronal somata of hippocampal brain slices. All experiments were conducted in artificial cerebrospinal fluid (aCSF) with 0 Mg^2+^ and 10 μM Bicuculline. (**A**) **Left**, representative image of GCaMP expression in control mouse hippocampal brain slice, with insets just before and during a Ca^2+^ event. **Right**, representative trace of baseline activity from a CA1 pyramidal neuron cell body (denoted by blue arrow); (**B**) **Left**, representative image of GCaMP expression in MIF-treated mouse hippocampal brain slice, with insets just before and during a calcium event. **Right**, representative trace from a CA1 pyramidal neuron cell body (denoted by blue arrow); (**C**–**E**) Average data of spontaneous events from two to three slices per condition, in four control mice and in four MIF-treated mice; (**C**) frequency (events/min); (**D**) Amplitude (*ΔF/F*); (**E**) Half-width (sec); *p* values based on Mann-Whitney test are shown in panels C,D and E. * denotes significance at *p* < 0.05.

The effect of MIF exposure on Ca^2+^ signals was assessed in CA1 pyramidal neuron soma, in acute mouse brain slices. Mice were stereotactically injected with a genetically encoded calcium indicator, GCaMP6f, into the CA1 region of the hippocampus and co-injected with either saline or 200 ng of recombinant MIF peptide (rMIF). Two weeks later, 300 μm thick live hippocampal slices were obtained and imaged for Ca^2+^ events in CA1 pyramidal neurons. Robust GCaMP6f expression was observed in CA1 pyramidal neuron soma, and their apical dendrites in stratum radiatum in both saline and MIF-injected animals (Figure 1A,B). To optimize conditions for observing Ca^2+^ activity in CA1 pyramidal neurons, hippocampal slices were perfused with Mg^2+^ free extracellular solution and 10 μM bicuculline. While the incidence of baseline activity under these conditions was similar in saline and MIF-treated mice (two of 21 slices for saline, two out of 26 slices for MIF), Ca^2+^ signal kinetics were altered in MIF-injected mice (Supplementary movies S1 & 3). Specifically, MIF-treated animals displayed a significantly higher frequency of Ca^2+^ events when compared to saline controls (Mann–Whitney test, *p* = 0.035, *n* = 4 mice per condition and two to three slices per mouse, per condition) (Figure 1C). However, the amplitude and half-width of Ca^2+^ events in MIF-injected mice was not significantly different from controls (Mann-Whitney test, *p* = 0.26 for amplitude and *p* = 0.38 for half width) (Figure 1D,E). These data are summarized in Table 1, and suggest exposure to MIF increases the frequency of baseline calcium activity in CA1 pyramidal neurons of adult mice.

### 2.2. MIF Does Not Significantly Alter N-methyl-d-aspartate NMDA + D-Serine Evoked Response in CA1 Layer Neuronal Somata

**Figure 2:**
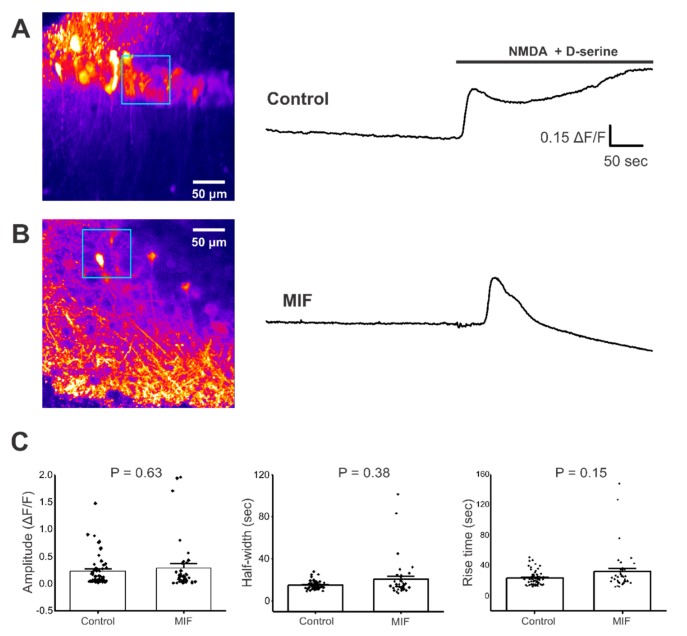
NMDA + D-serine response in CA1 neuronal somata of hippocampal brain slices. All experiments were conducted in aCSF with 0 Mg^2+^ and 10 μM Bicuculline. (**A**) **Left**, Representative image of GCaMP expression in the CA1 layer of hippocampal brain slices from control mice (blue square represents ROI). **Right**, representative trace from ROI, with and without NMDA + D-serine application; (**B**) **Left**, Representative image of GCaMP expression in the CA1 layer of hippocampal brain slices from MIF mice (blue square represents ROI). **Right**, representative trace from ROI with and without NMDA + D-serine application; (**C**) Average amplitude, half-width and rise time data of NMDA + D-serine response from two to four slices each, from eight control mice and four MIF-treated mice. *p* values based on Mann-Whitney test are shown for graphs in C.

Since induction of basal activity required conditions (0 Mg^2+^ and bicuculline) that enhance *N*-methyl-d-aspartate (NMDA) channel activity, we sought to assess the effect of MIF on NMDA channel function in hippocampal CA1 pyramidal neurons. Pharmacologically-evoked Ca^2+^ responses with bath application of 300 μM NMDA + 100 μM D-serine were measured in hippocampal CA1 pyramidal neurons from both saline and MIF-exposed mice, and the response amplitude, half-width, and rise time was quantified. NMDA + D-serine elicited a single robust Ca^2+^ response in both conditions (Supplementary movies S2 & S4). Responses were sustained and longer than baseline activity Ca^2+^ events (Figure 2A,B). For regions of interest (ROIs) located in the CA1 layer of the hippocampus, the amplitude, half-width, and rise time of NMDA + D-serine-evoked Ca^2+^ responses were not significantly different between groups (Mann-Whitney test, *p* = 0.63 for amplitude; *p* = 0.38 for half width; *p* = 0.15 for rise time; *n* = 2 to 3 slices from each mouse in both conditions) (Figure 2C). These data suggest that MIF had no significant effect on the Ca^2+^ responses to NMDA + D-serine in the CA1 cell layer. 

### 2.3. NMDA + D-Serine Response in Apical Dendrites of CA1 Neurons Is Altered by MIF

**Figure 3:**
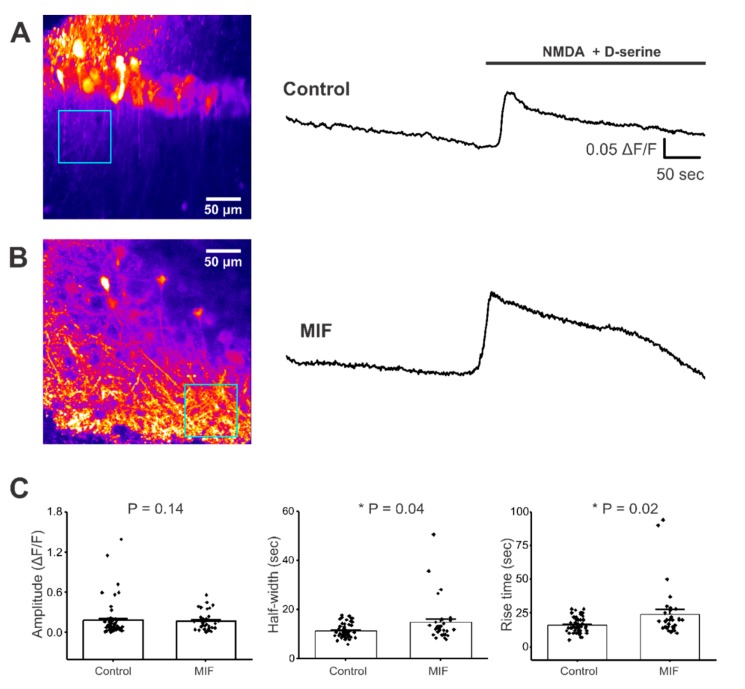
NMDA + D-serine response in CA1 stratum radiatum neuronal processes of hippocampal brain slices. All experiments were conducted in aCSF with 0 Mg^2+^ and 10 μM Bicuculline. (**A**) **Left**, Representative image of GCaMP expression in the stratum radiatum of hippocampal brain slices from control mice (blue square represents ROI). **Right**, representative trace from ROI, with and without NMDA + D-serine application; (**B**) **Left**, Representative image of GCaMP expression in the stratum radiatum of hippocampal brain slices from MIF-treated mice (blue square represents ROI). **Right**, representative trace from ROI, with and without NMDA + D-serine application; (**C**) Average amplitude, half-width and rise time data of NMDA + D-serine response from two to four slices each, from eight control mice and four MIF-treated mice. *p* values based on Mann-Whitney test are shown for graphs in C. * denotes significance at *p* < 0.05.

Distinct subtypes of NMDA receptors are expressed in the pyramidal neuron cell bodies and apical dendritic processes of hippocampal neurons, located within stratum radiatum [49]. GCaMP6f expression was observed throughout these apical dendrites in CA1 stratum radiatum in saline and MIF-treated mice (Figure 3A,B). To determine if MIF alters kinetics of NMDA + D-serine responses, specifically in the stratum radiatum processes, we analyzed Ca^2+^ responses in this subregion. For ROIs located in the stratum radiatum, MIF had no significant effect on the amplitude of Ca^2+^ responses, but MIF significantly increased both half-width and rise time, of Ca^2+^ responses, compared to saline controls (Mann-Whitney test, *p* = 0.14 for amplitude; *p* = 0.04 for half width; *p* = 0.02 for rise time) (Figure 3C). These data, summarized in Table 2, demonstrate a prolonged Ca^2+^ response in CA1 stratum radiatum, following NMDA + D-serine application in MIF-treated animals, when compared to saline controls. This finding suggests that MIF specifically alters the kinetics of NMDA receptor function in CA1 pyramidal cell apical dendrites, without an effect on the NMDA kinetics of CA1 pyramidal neuron somata.

## 3. Discussion

A direct effect of MIF on hippocampal neuronal function has not been previously explored. In this study, we demonstrate for the first time, that MIF alters baseline functional properties of hippocampal CA1 pyramidal neurons (Figure 1), and changes the kinetics of pharmacologically-evoked NMDA responses in the apical dendrites of these neurons (Figure 3). MIF is known to be involved in several inflammatory and trophic responses, via its role in modulating signaling mechanisms that are regulated by TLR4, p53 and CD74 signaling [11,28,29,30,31,32,33,34,35,36,37,38,39,40]. While there is evidence showing the involvement of MIF in inflammatory pathways, as well as evidence for MIF effects on peripheral neurons, this is the first study to show that MIF influences cortical neurons. These findings have widespread implications for a range of neurological and neuropathological functions. 

Considering that MIF is elevated relatively soon after a TBI, it is possible that MIF might be directly involved in single-cell and circuit-level alterations observed following a brain insult. We found that a single in vivo injection of 200 ng recombinant MIF was sufficient to induce long lasting (~two weeks) effects on neuronal activity. A single exposure of the mouse hippocampus to exogenous MIF led to significant increases in the baseline frequency of Ca^2+^ events in mouse CA1 layer pyramidal neurons as late as two weeks after exposure (Figure 1 and Table 1). This suggests the possibility that injury-induced increases in MIF secretion by astrocytes and neurons may be sufficient to initiate processes that induce long-term changes in neuronal function. Interestingly, MIF increased Ca^2+^ event frequency with no effects on the amplitude and half-width of baseline Ca^2+^ events (Figure 1), suggesting that MIF specifically triggers a change in the membrane excitability of CA1 pyramidal neurons. Since biophysical properties of Kv1 voltage-gated potassium channels in macrophages can be modified by activation or suppression of immune components [50], and because MIF is an important immune mediator [11], our study is consistent with the notion that alterations in immune signaling can affect neuronal excitability and/or function. Another study shows that MIF increases firing frequency of dorsal root ganglion by almost three-fold, within ~3 min, further supporting the role of early and direct interactions between MIF and Kv channels [45]. Considering that neuronal insults such as TBI increase seizure susceptibility, it is enticing to speculate that changes in MIF-induced baseline excitability of hippocampal neurons, via an interaction with Kv channels, might directly promote epileptogenic activity in the hippocampus. Future studies will assess the epileptogenic effects of stimulating and blocking MIF signaling. 

MIF modulates NMDA kinetics, another excitatory component of hippocampal neurons that has been implicated in hyperexcitability following brain insults such as TBI. Although NMDA + D-serine-evoked Ca^2+^ responses in CA1 pyramidal neuron cell bodies only displayed a trend towards an increase in half-width and rise time, (Figure 2 and Table 2), NMDA + D-serine significantly increased the amplitude, half-width, and rise time of Ca^2+^ responses in the apical dendrites of these CA1 pyramidal cells (Figure 3 and Table 2). Since increases in half-width and rise time reached significance in these apical dendrites, but not the CA1 somata, it appears that stratum radiatum processes are more vulnerable to MIF-associated changes in NMDA function. In models of epileptogenesis, including brain insult models, a major contributor to epileptiform activity is ectopic mossy fiber sprouting from the granule cells of the dentate gyrus to hippocampal pyramidal cells. This aberrant sprouting is, at least in part, dependent on NMDA activity [51,52]. Although we have examined apical dendrites in stratum radiatum of CA1 and not CA3, the data from the current study showing a direct influence of MIF on NMDA activity suggests a mediation of NMDA function in hippocampal pyramidal neurons. It is possible that MIF induces alteration to the stoichiometric composition and/or subcellular localization of NMDA receptors. Since specific subtypes of NMDA receptors display completely different biophysical properties and desensitization kinetics [53,54], one may observe a differential response in neuronal soma versus processes. Future studies will dissect out these possibilities by assessing NMDA trafficking and assembly, and whether these effects arise from extracellularly secreted astrocytic, neuronal, or other sources of MIF. 

The finding that a single microinjection of MIF is sufficient to modulate the kinetics of both, baseline Ca^2+^ activity and pharmacologically-evoked Ca^2+^ responses in CA1 layer somata and their apical dendrites, respectively, suggests that MIF can modulate more than one functional property of pyramidal neurons. Since this is a novel modulatory role, little is known about the mechanism by which MIF drives the observed effects on Ca^2+^ signaling in hippocampal neurons. Another unanswered question, is whether or not the introduction of recombinant MIF stimulates or inhibits the production of endogenous MIF. Therefore, the acute/chronic nature of MIF exposure using this method is unknown. While it is possible that MIF is driving these effects indirectly via astrocytes, the time course of response is suggestive of a sustained effect on neurons, even at two weeks after MIF was microinjected into the hippocampus. Subsequent studies will further investigate acute effects of MIF exposure on neurons, as well as direct interactions with extracellular membrane proteins, including NMDA expressed on hippocampal neurons. 

## 4. Materials and Methods 

### 4.1. Mice

Mouse care and experimental procedures were approved by the Texas A&M University Institutional Animal Care and Use Committee (9^th^ April 2017) and the animal use protocol approval number is 2017-0053. Adult (2–3 month) male C57BL/6 wildtype mice were used. Mice were group housed with food and water available ad libitum and kept in a temperature-controlled environment with a 12:12 h light:dark cycle.

### 4.2. Experimental Groups

This study uses two experimental groups of mice. Control mice were injected with 0.9% saline vehicle into the CA1 region of the hippocampus, while experimental mice were injected with 200 ng recombinant MIF peptide in 0.9% saline into the CA1 region of the hippocampus. Additionally, both control and MIF-injected mice received an injection of AAV.Syn.GCaMP6f.WPRE.SV40 into the hippocampus in order to detect calcium events. Only one surgery per animal was performed with both injections (Saline + AAV or MIF + AAV) being made simultaneously. 

### 4.3. Stereotaxic Microinjections for In Situ Hippocampal Neuron Imaging

All surgical procedures were done under general anesthesia using isoflurane (induction 5%, maintenance 1–2.5% vol/vol) as previously reported. Anesthesia was monitored continuously and adjusted as needed. Following induction, mice were placed in a stereotaxic frame (David Kopf Instruments, Tujunga, CA, USA) with their head fixed via blunt ear bars and their nose placed in a nose cone which delivers the anesthetic (Kent Scientific, Torrington, CT, USA). The surgical incision site was sterilized by three cleanings with 10% povidone iodine and 70% ethanol. Skin incisions were made and then followed by craniotomies of 2–3 mm above the right parietal cortex using a small steel burr (Fine Science Tools, Foster City, CA, USA) powered by a high-speed drill (K.1070; Foredom, Bethel, CT, USA). Saline (0.9%) was applied to the drilling site to reduce heat, and to keep the incision from drying out. Unilateral injections of AAV + MIF-, or saline injections, were performed using a stereotaxic apparatus to guide the placement of beveled glass pipettes (World Precision Instruments, Sarasota, FL, USA), into the right hippocampus (2 mm posterior to bregma, 1.5 mm lateral to midline, and 1.6 mm from the pial surface). For the injections, 1.5 μL pAAV.Syn.GCaMP6f.WPRE.SV40 (titer of 1 × 10^13^ gc/mL, Addgene, Cambridge, MA, USA) plus 200 ng of saline, or human MIF peptide (Sino Biological) were injected using a syringe pump (PicoPump11; Harvard Apparatus, Holliston, MA, USA). Following injection, glass pipettes were left in place for 10 min, and then gradually withdrawn. Nylon sutures (external 5-0 nylon) were used to close the surgical wound, after which animals were allowed to recover in a cage placed on a low-voltage heating pad. No analgesics were used. Mice were sacrificed at 14 d after surgery for imaging.

### 4.4. Brain Slice Preparation and Confocal Ca^2+^ Imaging

Brain slice imaging was performed as previously reported. Adult mouse brain coronal slices were cut at 300 μm thickness using a vibratome (Microslicer dtk-1000, Ted Pella, Redding, CA, USA). Slicing solution contained (mM): 194 Sucrose, 30 NaCl, 4.5 KCl, 1.2 NaH_2_PO_4_, 26 NaHCO_3_, 10 d-glucose, 1 MgCl_2_, saturated with 95% O_2_ and 5% CO_2_. Slices were incubated in an artificial cerebrospinal fluid recording solution (aCSF) for 30 min at 34 °C, then moved to a room temperature for imaging. A CSF solution contained (mM): 126 NaCl, 2.5 KCl, 1.24 NaH_2_PO_4_, 1.3 MgCl_2_, 2.4 CaCl_2_, 26 NaHCO_3_, 10 d-glucose. Imaging was performed using a confocal microscope (Fluoview 1000, Olympus, Center Valley, PA, USA) with a 40× water-immersion objective (NA 0.8). We used the 488-nm line of a krypton-argon laser to excite GCaMP6f. For imaging Ca^2+^ signals, slices were focused to a plane where cell bodies of CA1 pyramidal neurons and their processes were clearly demarcated. The imaging frame was clipped in order to achieve a one frame per second (fps) sampling rate. For all experiments baseline activity was measured in a zero Mg^2+^ aCSF + 10 μM Bicuculline (Abcam, Cambridge, MA, USA) recording buffer for 300 s, then followed by bath application of 300 μM NMDA (Sigma, St. Louis, MO, USA) + 100 μM D-serine (Alfa Asar, Haverhill, MA, USA) for 300 s. Solutions and drugs were bath perfused at 2 mL/min using a peristaltic pump. 

### 4.5. Data Analysis 

Image analysis was performed using ImageJ v1.52e (NIH, Bethesda, MA, USA). Ca^2+^ events were analyzed using MiniAnalysis v6.0.7 (Fort Lee, NJ, USA). Image time series (*t*-series) files were adjusted to increase brightness in order to visualize all neuronal somata. Three square ROIs (75 μm × 75 μm) were drawn in both the CA1 cell layer and stratum radiatum in such a way that the ROIs were equidistant and did not overlap. *t*-series were then closed and reopened with default brightness/contrast. Mean gray value of each ROI for all image frames was extracted and then converted to *ΔF/F* values. Baseline *F* was determined from 10 s periods without transients/events. For baseline event analysis ROIs were drawn around individual cells, and only cells with one or more Ca^2+^ events in the absence of NMDA + D-serine application were selected. For Ca^2+^ event analysis, frequency was measured as the total number of Ca^2+^ events divided by the recording time, and expressed as events/min. Amplitude was measured as the magnitude of change in fluorescence (*ΔF/F)* from baseline to event peak. Rise time was measured as the time in seconds for an individual Ca^2+^ event to reach its peak amplitude from baseline. Half-width was measured as the time for an individual Ca^2+^ event to reach 50% of its peak amplitude from baseline. 

### 4.6. Statistical Analyses

All data and statistical analyses were performed using OriginLab 2019 (Northhampton, MA, USA). Data presented are mean ± S.E.M. For each data set, normality was first determined in Origin using the Shapiro–Wilk test. Non-normally distributed data were analyzed via Mann–Whitney tests. Data was considered to be significantly different at *p* < 0.05. For each analysis performed exact *p*-values are reported.

## Figures and Tables

**Table 1:** Summary of spontaneous activity.

Condition	% of Slices w/Baseline Activity	Mean Frequency (events/min)	Mean Amplitude (*ΔF/F*)	Mean Half-Width (sec)
Control	10	1.667 ± 0.68	0.328 ± 0.05	1.6274 ± 0.167
MIF	8	3.467 ± 0.99 *	0.215 ± 0.05	1.9250 ± 0.277

Values presented as Mean ± SEM. * denotes significance at *p* < 0.05.

**Table 2:** Summary of NMDA Responses.

Condition	% of Slices Responded	Mean Amplitude (*ΔF/F*)		Mean Half-Width (sec)		Mean Rise Time (sec)	
		CA1 Cell Layer	Stratum Radiatum	CA1 Cell Layer	Stratum Radiatum	CA1 Cell Layer	Stratum Radiatum
Control	55	0.231 ± 0.04	0.180 ± 0.18	14.962 ± 0.53	11.370 ± 0.39	23.333 ± 1.29	16.211 ± 0.74
MIF	50	0.289 ± 0.08	0.177 ± 0.02	20.445 ± 2.98	14.786 ± 1.52 *	31.974 ± 4.48	24.303 ± 3.36 *

Values presented as Mean ± SEM. * denotes significance at *p* < 0.05.

## Supplementary Materials

The following are available online at https://www.mdpi.com/1422-0067/21/1/276/s1, Movie S1: Spontaneous Ca^2+^ events in the hippocampus of a saline injected mouse. Representative movie showing spontaneous activity of CA1 pyramidal neurons in the hippocampus of acute brain slices from a saline injected mouse. Slices were bath perfused with aCSF containing zero Mg^2+^ and 10 µM bicuculline. Note the spontaneous activity in both neuronal soma (white arrowheads) and neuronal processes (yellow arrowheads). Movie S2: NMDA-induced Ca^2+^ response in the hippocampus of a saline injected mouse. Representative movie showing a Ca^2+^ response to bath applied NMDA + D-serine in the hippocampus of acute brain slices from a saline injected mouse. Slices were bath perfused with aCSF containing zero Mg^2+^ and 10 µM bicuculline. Note the slow and sustained Ca^2+^ response to NMDA + D-serine in both neuronal soma (white arrowheads) and neuronal processes (yellow arrowheads). Movie S3: Spontaneous Ca^2+^ events in the hippocampus of a MIF injected mouse. Representative movie showing spontaneous activity of CA1 pyramidal neurons in the hippocampus of acute brain slices from a MIF injected mouse. Slices were bath perfused with aCSF containing zero Mg^2+^ and 10 µM bicuculline. Note the robust increase in spontaneous activity after a single *in vivo* injection of recombinant MIF peptide in both neuronal soma (white arrowheads) and neuronal processes (yellow arrowheads). Movie S4: NMDA-induced Ca^2+^ response in the hippocampus of a MIF injected mouse. Representative movie showing a Ca^2+^ response to bath applied NMDA + D-serine in the hippocampus of acute brain slices from a MIF injected mouse. Slices were bath perfused with aCSF containing zero Mg^2+^ and 10 µM bicuculline. Note the more robust and sustained Ca^2+^ response to NMDA + D-serine in both neuronal soma (white arrowheads) and neuronal processes (yellow arrowheads) after a single *in vivo* injection of recombinant MIF peptide into the mouse hippocampus.

## Abbreviations

MIF	Macrophage migration inhibitory factor
TBI	Traumatic brain injury
CNS	Central nervous system
CD	Cluster of differentiation
CLIP	Class II-associated invariant peptide
DAMP	Damage associated molecular pattern
PAMP	Pathogen associated molecular pattern
PRR	Pattern recognition receptor
NOD	Nucleotide-binding oligomerization domain-like
NLR	Nucleotide-binding oligomerization domain-like (NOD) receptor
NFkB	Nuclear factor kappa-light-chain-enhancer
TLR	Toll-like receptor
NMDA	*N*-methyl-d-aspartate
AMPA	α-amino-3-hydroxy-5-methyl-4-isoxazolepropionic acid
AngII	Angiotensin II
DRG	Dorsal root ganglion
PNS	Peripheral nervous system
IFN-γ	Interferon gamma
PTE	Post-traumatic epilepsy
GECI	Genetically encoded calcium indicator
aCSF	Artificial cerebrospinal fluid recording buffer
mRNA	Messenger ribonucleic acid
